# Functional analysis of *Paracoccidioides brasiliensis* 14-3-3 adhesin expressed in *Saccharomyces cerevisiae*

**DOI:** 10.1186/s12866-015-0586-2

**Published:** 2015-11-04

**Authors:** Patricia Akemi Assato, Julhiany de Fátima da Silva, Haroldo Cesar de Oliveira, Caroline Maria Marcos, Danuza Rossi, Sandro Roberto Valentini, Maria José Soares Mendes-Giannini, Cleslei Fernando Zanelli, Ana Marisa Fusco-Almeida

**Affiliations:** Laboratório de Micologia Clínica - Núcleo de Proteômica – Faculdade de Ciências Farmacêuticas- Unesp - Campus Araraquara, Rodovia Araraquara - Jaú Km 1, 14801-902 Araraquara, SP Brazil; Laboratório de Biologia Molecular – Faculdade de Ciências Farmacêuticas- Unesp - Campus Araraquara, Rodovia Araraquara - Jaú Km 1, 14801-902 Araraquara, SP Brazil

**Keywords:** *Paracoccidioides brasiliensis*, 14-3-3 protein, Adhesion, Adhesin

## Abstract

**Background:**

14-3-3 proteins comprise a family of eukaryotic multifunctional proteins involved in several cellular processes. The Pb14-3-3 of *Paracoccidioides brasiliensis* seems to play an important role in the *Paracoccidioides*-host interaction. *Paracoccidioides brasiliensis* is an etiological agent of paracoccidioidomycosis, which is a systemic mycosis that is endemic in Latin America. In the initial steps of the infection, *Paracoccidioides* spp. synthetizes adhesins that allow it to adhere and invade host cells. Therefore, the aim of this work was to perform a functional analysis of Pb14-3-3 using *Saccharomyces cerevisiae* as a model.

**Results:**

The functional analysis of Pb14-3-3 was performed in *S. cerevisiae,* and it was found that Pb14-3-3 partially complemented *S. cerevisiae* proteins Bmh1p and Bmh2p, which are recognized as two yeast 14-3-3 homologues. When we evaluated the adhesion profile of *S. cerevisiae* transformants, Pb14-3-3 acted as an adhesin in *S. cerevisiae*; however, Bmh1p did not show this function. The influence of Pb14-3-3 in *S. cerevisiae* ergosterol pathway was also evaluated and our results showed that Pb14-3-3 up-regulates genes involved in ergosterol biosynthesis.

**Conclusions:**

Our data showed that Pb14-3-3 was able to partially complement Bmh1p and Bmh2p proteins in *S. cerevisiae;* however, we suggest that Pb14-3-3 has a differential role as an adhesin. In addition, Pb-14-3-3 may be involved in *Paracoccidioides* spp. ergosterol biosynthesis which makes it an interest as a therapeutic target.

## Background

*Paracoccidioides brasiliensis* and *Paracoccidioides lutzii* are the etiological agents of paracoccidioidomycosis, an endemic, systemic mycosis in Latin America, with the highest prevalence in Brazil (80 % of cases), where the Southeast and South regions report most of the cases [1, 2]. It is the eighth highest cause of death among infectious and parasitic diseases and has the highest mortality rate, up to 59 %, between systemic mycosis in Brazil in endemic areas [3–5].

The infection occurs through the inhalation of conidia of the mycelial form. Once inside the host, the fungus undergoes a transition to the yeast form, also known as the parasitic form, via temperature stimulation [6]. However, the establishment of the infection depends on several factors, such as the host immune system, the ability of the fungus to evade it and establish itself in the hostile environment provided by the host. In this way *Paracoccidioides* spp. synthesize several substances that may cause damage in the host cells and assist in colonization [7–10].

An important feature in the host-pathogen interaction is the adhesion process, which contributes to pathogen colonization, dissemination and evasion of the host immune system [11, 12]. Several adhesins that allow the fungus to bind the host extracellular matrix (ECM) have already been described for *Paracoccidioides* spp. [9]. Among these, 14-3-3 protein plays an important role in *Paracoccidioides*-host interaction.

The 14-3-3 protein from *P. brasiliensis*, termed Pb14-3-3, belongs to the 14-3-3 protein family, and it is composed of approximately 30 kDa acidic dimeric proteins that have already been described in all eukaryotes and are involved in many cellular processes [13–18].

The Pb14-3-3 protein was first described as an adhesin and a laminin ligand [19] and it was identified in *P. brasiliensis* extracellular vesicles [20]. Da Silva et al. [21] demonstrated using in vitro and in vivo models that during infection, an accumulation of this protein occurred in the fungal cell wall. Additionally, the recombinant protein promoted a decrease in the adhesion rate of *P. brasiliensis* to epithelial cells.

In a study conducted by de Oliveira et al. [22] the expression of adhesins genes and adhesion profile of both *P. brasiliensis* and *P. lutzii* were compared during interaction with mice and they observed that in both species Pb14-3-3 gene is up-regulated, showing that this protein plays an important role in the host-pathogen interaction in both *Paracoccidioides* species.

Recently, da Silva et al. [23] evaluated the pneumocytes response when treated with gp43 and Pb14-3-3. The cells exhibited the same profile of apoptosis signaling observed during *P. brasiliensis* infection, highlighting the importance of this protein during the interaction with the host.

*Saccharomyces cerevisiae* has two encoding genes, *BMH1* and *BMH2,* for 14-3-3 proteins (Bmh1p and Bmh2p) that are involved in innumerable processes, such as sporulation, ergosterol metabolism-related gene transcription and chitin synthesis [14, 24–28].

Although advances in the genetic manipulation of *P. brasiliensis* have been made, *Saccharomyces cerevisiae* is still extensively used for genetic studies, including functional analyses, due its ease of use and the wide range of available information. [29–34] Thus, we chose this yeast as our model to evaluate the role of the Pb14-3-3 and its relationship with the pathogenicity of *P. brasiliensis*.

## Results

### Primary sequence alignment of 14-3-3 proteins

**Fig. 1:**
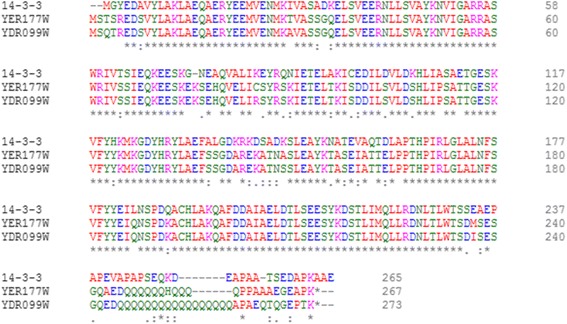
Primary sequence analysis. An “*” (*asterisk*) indicates positions that have a single, fully conserved residue; A “:” (*colon*) indicates conservation between groups with strongly similar properties - scoring > 0.5 in the Gonnet PAM 250 matrix; A “.” (*period*) indicates conservation between groups of weakly similar properties - scoring = < 0.5 in the Gonnet PAM 250 matrix. The code of colors according to their physicochemical properties are as follows: red, small and hydrophobic (AVFPMILW); blue, acidic (DE); magenta, basic (RK); and green, (STYHCNGQ)

As previously described, *S. cerevisiae* has two 14-3-3 isoforms, Bmh1p [GenBank: DAA07840.1] and Bmh2p [GenBank: DAA11946.1] [35]. Therefore, using the ClustalW2 amino acid sequence, an analysis was performed between them and Pb14-3-3 [GenBank: AAR24348.1], and a high identity was found among these proteins: Bmh1p and Pb14-3-3 presented an identity of 76 %, and Bmh2p and Pb14-3-3 presented an identity of 80 % (Fig. 1).

### Molecular cloning of Pb14-3-3 gene and *BMH1*

**Fig. 2:**
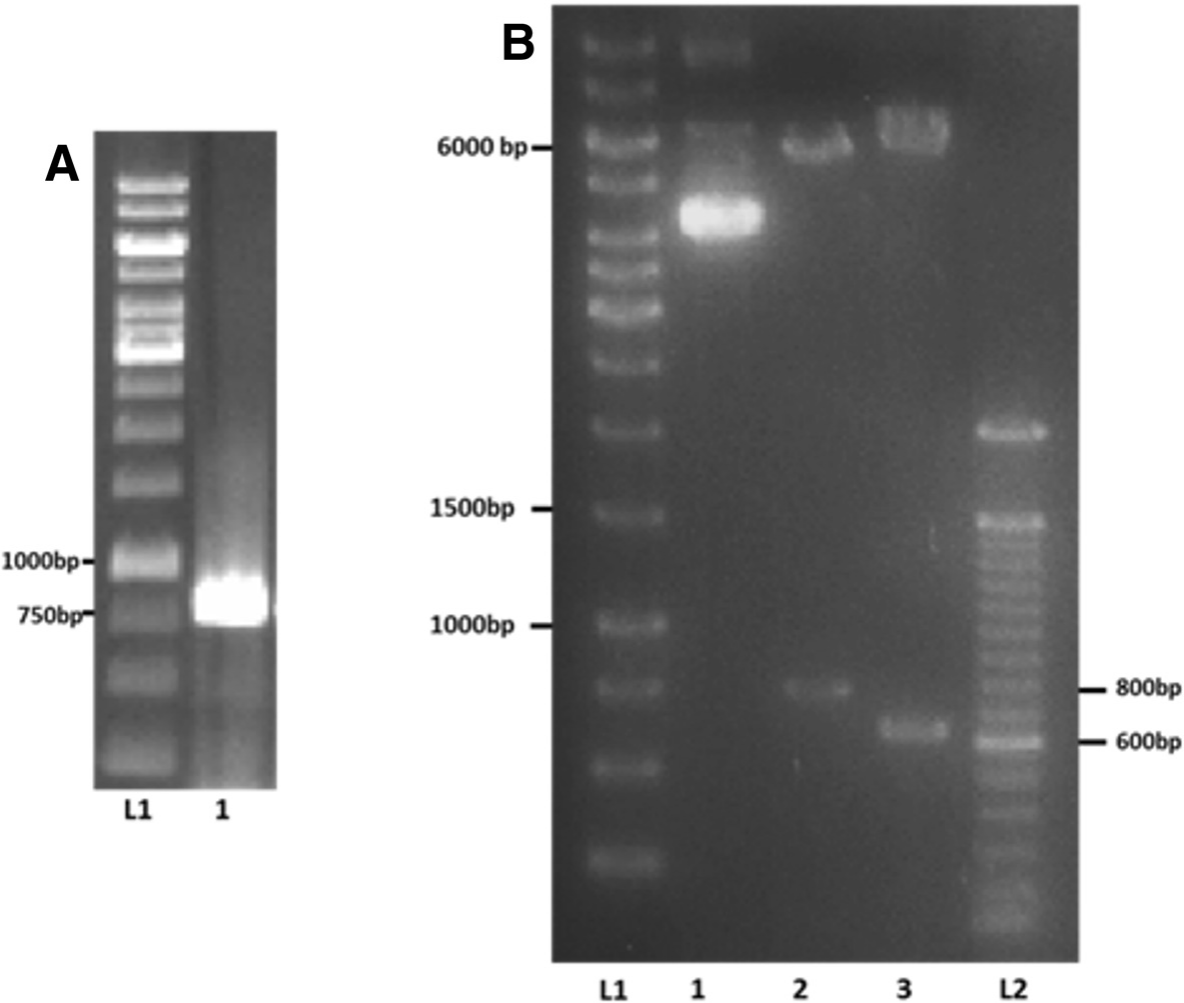
Agarose gel electrophoresis. **a** Confirmation of Pb14-3-3 ORF amplification: (L1) 1 kb ladder and (2) Pb14-3-3 ORF (798 bp). **b** Restriction diagnosis: (L1) 1 kb ladder; 1 – pYES-14-3-3, (2) restriction products using *BamH1* and *XhoI* (expected product 798 bp), and (3) restriction products using *BamHI* and *HindIII* (expected product 605 bp), (L2) 100 bp ladder

The heterologous expression of Pb14-3-3 in *S. cerevisiae* was carried out using *P. brasiliensis* cDNA to amplify the Pb14-3-3 ORF [GenBank: AY462124] (Fig. 2a), followed by cloning into pYES2 vector. As a control, these procedures were performed using *BMH1* ORF [GenBank: X66206.1] because it is the predominantly expressed isoform in *S. cerevisiae* [35]. After cloning confirmation (Fig. 2b) and sequence analysis, the obtained plasmids, pYES-14-3-3 and pYES-BMH1, and the empty plasmid, pYES, were transformed into *wild type* (wt), *Δbmh1* and *Δbmh2 S. cerevisiae* strains using the lithium acetate method for yeast transformation. The positive transformants were selected on SD-URA (synthetic defined medium without uracil) plates.

### Complementation assay

**Fig. 3:**
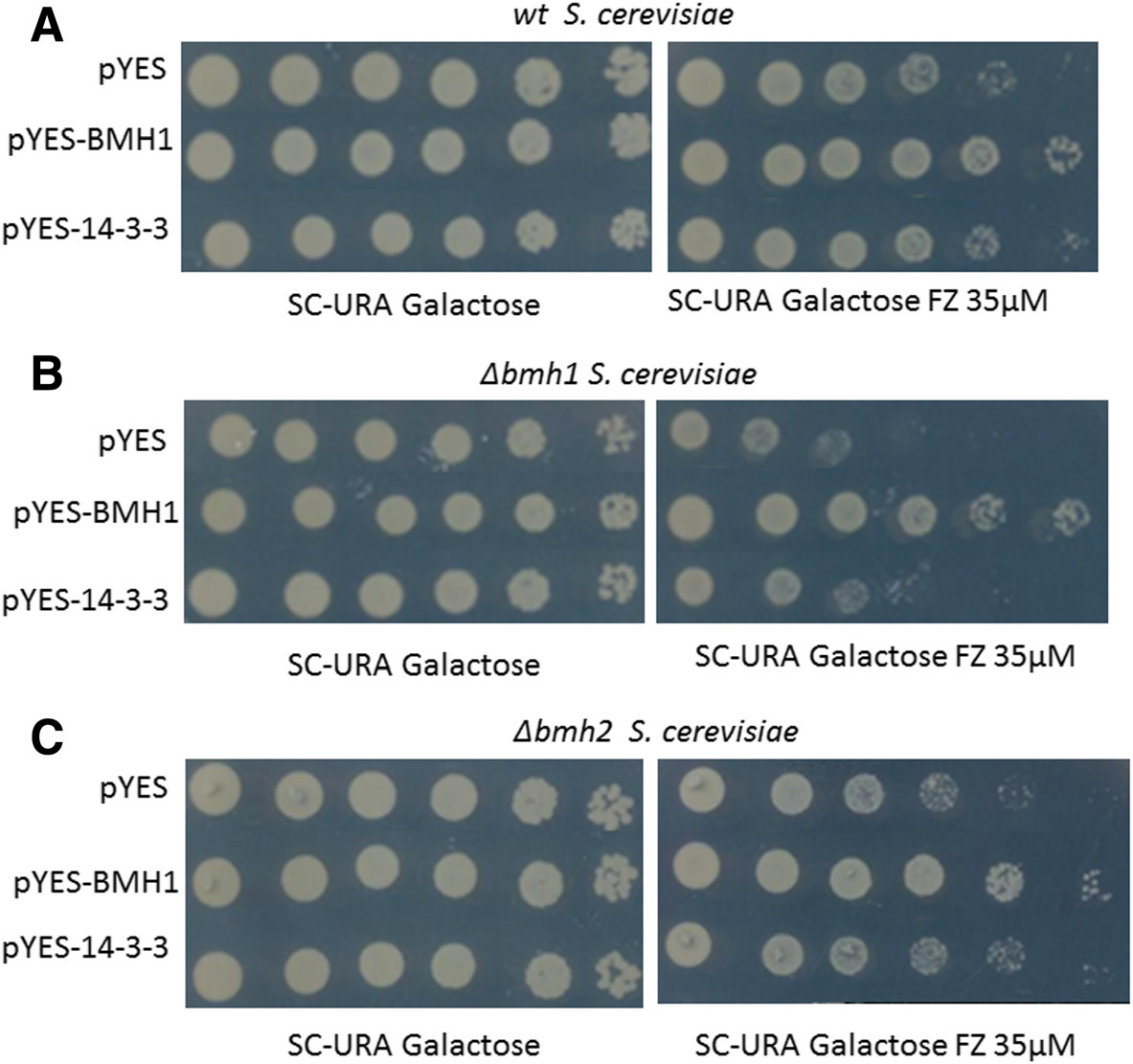
Evaluation of complementation. A spot assay was used to evaluate susceptibility to fluconazole 35 μM in *wt* (**a**), *Δbmh1* (**b**) and *Δbmh2* (**c**) *S. cerevisiae* transformants. There was a decreased sensitivity of transformants pYES-14-3-3 and pYES-BMH1 compared with the empty vector transformant (pYES). As a growth control, the transformants were also spotted in SD-URA without fluconazole

To evaluate the ability of Pb14-3-3 to complement Bmh1p or Bmh2p function in the *Δbmh1* and *Δbmh2 S. cerevisiae* mutants or to improve the functions of these proteins in the *wt S. cerevisiae*, we performed a spot test using fluconazole (FZ) 35 μM as described in the Materials and Methods. The Pb14-3-3 promoted a decrease in fluconazole susceptibility, and a higher complementation was observed in the *Δbmh2 S. cerevisiae* mutant, probably due to the higher identity of Pb14-3-3 with Bmh2p from *S. cerevisiae* (Fig. 3).

In addition, as fluconazole acts in ergosterol biosynthesis, there is evidence that Pb14-3-3 may be involved in ergosterol biosynthesis, as found with *S. cerevisiae* Bmh1/2 proteins [26, 36].

### Expression analysis of genes related to ergosterol pathway

In order to test our hypothesis, we evaluated the expression of genes involved in ergosterol pathway by Real Time PCR. Bmh1p and Bmh2p influence in ergosterol pathway have already been described and downregulation of *ERG1*, *ERG11*, *ERG28* and *HES1* genes were observed in *S. cerevisiae BMH1/2* mutants [26]*.* In this sense, those genes were chosen to evaluate the possible role of Pb14-3-3 in ergosterol pathway.

**Fig. 4:**
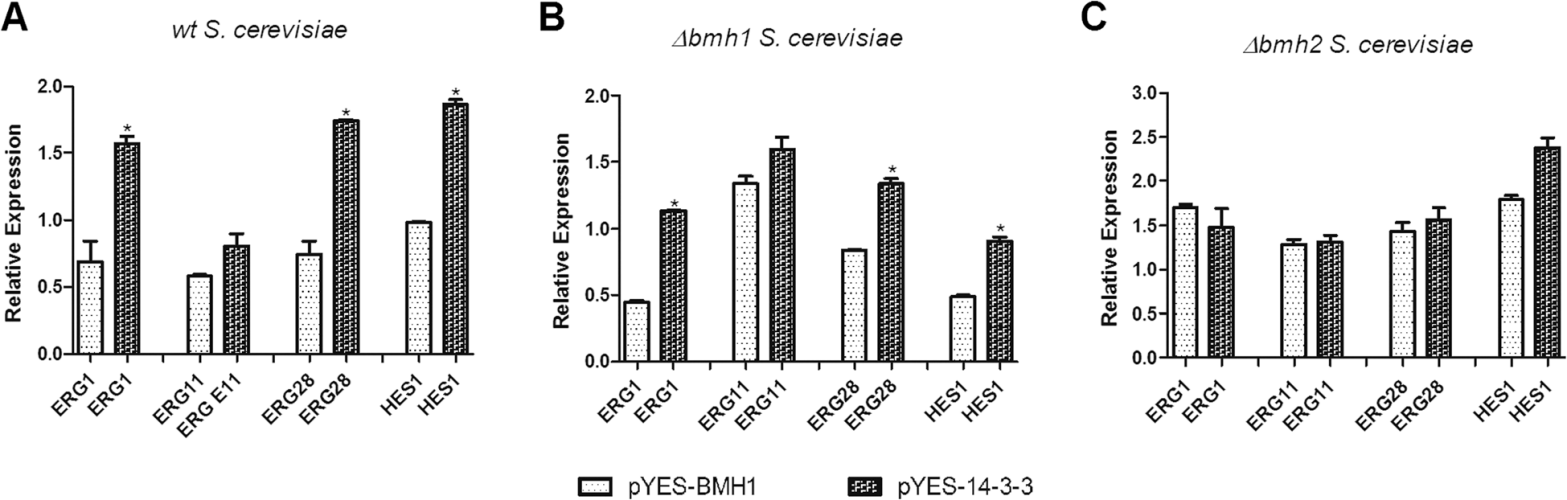
Relative gene expression of genes involved in ergosterol pathway. Relative gene expression of *ERG1*, *ERG11*, *ERG28* and *HES1* in (**a**) *wt S. cerevisiae* transformed with pYES-BMH1 and pYES-14-3-3, (**b**) *Δbmh1 S. cerevisiae* transformed with pYES-BMH1 and pYES-14-3-3 and (**c**) *Δbmh2 S. cerevisiae* transformed with pYES-BMH1 and pYES-14-3-3. (*) Indicates a statistically significant difference in expression level, *p* < 0,05. The graph show the normalized gene expression relative to *wt, Δbmh1* and *Δbmh2 S. cerevisiae* transformed with pYES (empty vector), respectively

An increase of expression of all evaluated genes was observed in the three *S. cerevisiae* transformants containing pYES-BMH1 and pYES-14-3-3 when compared to the *S. cerevisiae* pYES.transformants (Fig. 4). The expression analysis showed an increased expression of all genes in the *wt* and *Δbmh1 S. cerevisiae* containing pYES-14-3-3 when compared to the pYES-BMH1 transformants, with a significant increase for *ERG1*, *ERG28* and *HES1*. In *Δbmh2 S. cerevisiae* transformed with pYES-14-3-3 a slight increase is observed for *ERG28* and *HES1* genes. These data demonstrate that Pb14-3-3 is also involved in ergosterol pathway by altering the expression of these genes.

In addition, it seems like Bmh1p has a higher influence in this pathway, once *ERG1*, *ERG11, ERG28* and *HES1* showed a higher expression in *Δbmh2 S. cerevisiae* containing pYES-BMH1, which have the *BMH1* gene and the plasmid, than *Δbmh1 S. cerevisiae*, which expression of Bmh1p occurs only through the plasmid.

### Adhesion assay

Pb14-3-3 was identified in our group as an adhesin, hence, we performed an adhesion assay to evaluate the ability of Pb14-3-3 to induce *S. cerevisiae* adherence to epithelial cells (ATCC A549).

**Fig. 5:**
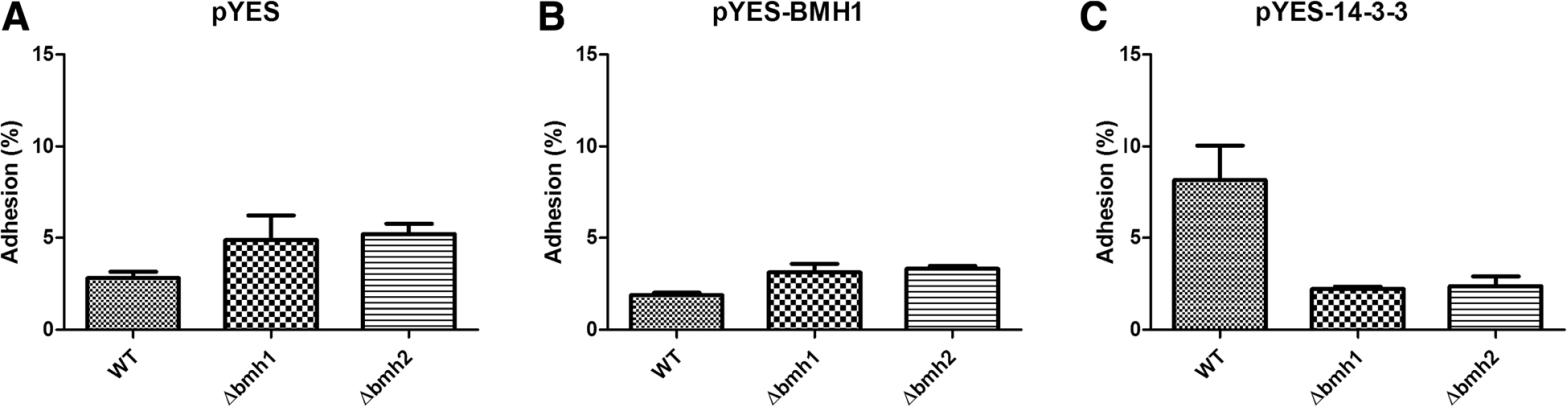
Adhesion assay. **a** Adhesion rate of *wt, Δbmh1* and *Δbmh2 S. cerevisiae* transformed with pYES (empty vector), (**b**) adhesion rate of *wt, Δbmh1* and *Δbmh2 S. cerevisiae* transformed with pYES-BMH1 and the (**c**) adhesion rate of *wt, Δbmh1* and *Δbmh2 S. cerevisiae* transformed with pYES-14-3-3. (*) Indicates a statistically significant difference in adhesion rate, *p* < 0,05

A significant increase in adhesion was only observed in *wt S. cerevisiae* transformed with pYES-14-3-3, showing that Pb14-3-3 acts as an adhesin even in a non-pathogenic fungus. However, Bmh1p did not promote the increase of the adhesion rate, suggesting a differential role of Pb14-3-3 (Fig. 5a).

In *Δbmh1* and *Δbmh2 S. cerevisiae* transformants with pYES14-3-3 or pYESBMH1 no increase of adhesion rate was observed, although it was unexpected, the Pb-14-3-3 may be acting in Bmh1p or Bmh2p primary functions in order to compensate the lack of missing genes, meanwhile *wt S. cerevisiae* possess both genes and Pb14-3-3 can be required for secondary functions, as adhesin (Fig. 5).

## Discussion

As with many pathogens, *Paracoccidioides* spp. adhesion to host cells is a crucial event in the establishment of disease and contributes to the colonization and invasion of host cells, as well as evasion from the host immune system [9, 11, 12]. Therefore, *Paracoccidioides* adhesins are important proteins to be studied because they might promote a better understanding of fungal pathogenesis and provide new targets for antifungals.

Several *Paracoccidioides* spp. adhesins have already been described, such as the 43 kDa glycoprotein, gp43, which is most studied *Paracoccidioides* spp. adhesin that binds to laminin and fibronectin and is also used as a serological marker [37–40]; glyceraldehyde-3-phosphate-dehydrogenase (GAPDH), enolase (ENO), triose phosphate isomerase (TPI), malate synthase (MLS), aconitase (ACO) and isocitrate lyase (ICL), which, in addition to their primary roles in the metabolism, are found in the fungal cell wall and are able to bind to different components of the ECM [41–49]; and the 30 kDa protein identified as the 14-3-3 protein, which stands out in *Paracoccidioides –* host interaction [21].

The 14-3-3 proteins comprise a family of small, dimeric and acidic proteins that are present in all eukaryotic cells [16, 18, 50]. The closest relation between 14-3-3 protein and fungus virulence was first described by Andreotti et al. [19], where they found an increase in the expression of a 30 kDa protein after animal reisolation and that this 30 kDa protein binds to laminin and it is able to inhibit *P. brasiliensis* adhesion and invasion in epithelial cells. Later, da Silva et al. [21] identified this protein as being related to a member of the 14-3-3 protein family and demonstrated that Pb14-3-3 accumulates in the fungal cell wall during infection in both in vitro and in vivo models.

As genetic manipulation of *P. brasiliensis* are still hard to accomplish [51], we decided to use *S. cerevisiae* as a model to improve our knowledge about Pb14-3-3. The double knockout of *BMH1* and *BMH2* genes are lethal [31], then we decided to evaluate Pb14-3-3 function in single mutant and wild type strains of *S. cerevisiae*, a strategy successfully used by Clapp et al. [52].

As the first description of Pb14-3-3 was as an adhesin, we performed an adhesion assay to evaluate the ability of Pb-14-3-3 to promote *S. cerevisiae* adhesion to pneumocytes. A significant increase in the adhesion rate was observed for *wt S. cerevisiae* transformed with pYES-14-3-3, but not with pYES-BMH1 and pYES. However, this was not observed in any transformed *S. cerevisiae* mutants (Fig. 5).

In this way, as *S. cerevisiae* is a non-pathogenic microorganism the adhesion process is not required to the yeast, therefore, when *Δbmh1* and *Δbmh2 S. cerevisiae* express Pb14-3-3, the protein is recruited to perform the primary functions of the missing gene, *BMH1* and *BMH2*, respectively, as it occurs with the Bmh1p and Bmh2 proteins [35]. However, in the *wt S. cerevisiae* transformants there is no need to supplement any function and the protein expressed through the plasmid perform secondary function and act as adhesin. Interesting, only *wt S. cerevisiae* pYES-14-3-3 showed an increase in adhesin rate compared to the *wt S. cerevisiae* pYES suggesting although the high identity, Pb14-3-3 may present different functions than Bmh1p, such as an adhesin.

Although 14-3-3 proteins are multifunctional proteins that are involved in several cellular processes [16, 18, 27, 28, 53], which may have influenced the adhesion of *S. cerevisiae*, the Pb14-3-3 role in adhesion process during *Paracoccidioides* spp. interaction with host cells in vitro and in vivo have already been demonstrated [21]. Additional role of conserved proteins in pathogenic organisms, including Paracoccidioides spp., during interaction with host cells has already been described. Enzymes from glycolytic pathway, tricarboxylic cycle and glyoxylate cycle are found in fungus cell wall during infection and are able to interact with ECM components, assisting in the fungus adherence [22, 49, 54].

In humans, this protein has been widely study as a target for drug development or as a biomarker [55–57] because it has been associated with neurodegenerative disease, such as Creutzfeldt-Jakob disease, Alzheimer’s disease [17, 24, 58, 59] and some cancer types [60, 61] .

However, the knowledge of the influence of 14-3-3 proteins in pathogenic fungi is still limited. *Candida albicans* encodes a single 14-3-3 gene, *BMH1* [62], which the expressed protein is involved in growth and filamentation that may affect the host-fungus interaction [63]. It was also recognized by *C. albicans* germ-tube-specific antibodies in the detection of invasive candidiasis, highlighting the potential as a biomarker for diagnosis [64].

The 14-3-3 proteins of some parasites have also been studied in regards their involvement in infectious diseases [65]. In *Trypanosoma brucei,* 14-3-3 proteins were related to cell motility, cytokinesis and the cell cycle [66, 67]; the 14-3-3 protein from *Toxoplasma gondii* seems to stimulate the host immune system and is a promising vaccine candidate [68, 69]. Several studies of 14-3-3 proteins from the genus *Ecchinococcus* suggest the importance of this protein in host-parasite interactions, where it can be involved in the regulation of cell proliferation, survival and invasion, as well as in the modulation of the host immune system [70–73]. The same behavior was also verified in 14-3-3 proteins from the genus *Schistosoma* and studies have been conducted to development of vaccines and diagnostic methods [74–78].

In *Aspergillus nidulans*, the expression of 14-3-3 protein was also identified and seems to be involved in hyphae morphogenesis as its overexpression promoted defects in the establishment of the germ tube and asexual development [79].

In this study, we also demonstrated that Pb14-3-3 could partially complement the functions of Bmh1p and Bmh2p by decreasing the susceptibility to fluconazole at 35 μM, showing a greater complementation in the mutant *S. cerevisiae Δbmh2*, which was expected because according to the amino acid alignment analysis, we observed a higher identity between Pb14-3-3 and Bmh2p than between Pb14-3-3 and Bmh1p.

In addition, it is well known that in azoles, such as fluconazole, the mechanism of action takes place in ergosterol biosynthesis by interrupting the conversion of lanosterol into 4,4-dimethylcholesta-8,14,24-trienol [80].

Thus, we hypothesized if Pb14-3-3 may be related to ergosterol biosynthesis due to the decreased sensibility of the *S. cerevisiae* strains transformed with pYES-14-3-3. To verify that, we performed an expression analysis of genes involved in ergosterol pathway, *ERG1*, *ERG11*, *ERG28* and *HES1*, which have already been described to be regulated by Bmh1p and Bmh2p [26].

The Pb14-3-3 promoted the up-regulation of all evaluated genes, demonstrating its involvement in ergosterol pathway in this model. In addition, the up-regulation promoted by Pb14-3-3 was higher than promoted by Bmh1p, this was specially observed for the genes *ERG1*, *ERG28* and *HES1 wt* and *Δbmh1 S. cerevisiae* transformants.

Ergosterol is the major sterol in fungi and it is an essential structural component of plasma membrane. Also, ergosterol acts in membrane permeability and in membrane-bound enzymes [81, 82]. In pathogenic fungus ergosterol is a microbe associated molecular pattern and because of the differences between ergosterol and human sterols it is widely study in the development of antifungal compounds [83–86].

The currently antifungals used in treatment of paracoccidioidomycosis, itraconazole and amphotericin B, have as target enzymes of ergosterol pathway, such as ERG11p, and ergosterol, respectively [87–91]. The resistance and effect of these antifungals in pathogenic fungi have been studied [92–94], Da Silva Neto *et al* [82], studied the transcriptional profile of *Paracoccidioides* treated with itraconazole and observed an up-regulation of genes from ergosterol biosynthesis.

The ergosterol biosynthesis is performed by genes from ERG family. *ERG1* and *ERG11* genes encode essential enzymes in the initial stages of ergosterol biosynthesis, acting in the conversion of squalene into squalene epoxide and of lanosterol into 4,4-dimethylcholesta-8,14,24-trienol, respectively. ERG28p seems to be a key enzyme in the ergosterol biosynthesis, once interacts with seven ergosterol biosynthetic enzymes, where the association with ERG11p, ERG27p, ERG25p and ERG6p are more closely and with ERG1p and ERG26p are less associated [95]. *HES1* is related to ergosterol biosynthesis and sterol transport [96, 97].

The role of *Paracoccidioides* spp. in adhesion process and this new role here described, in ergosterol biosynthesis, reinforce the importance of this protein in host-pathogen interactions and its potential as therapeutic target.

## Conclusion

These findings corroborate results presented in previous studies. Here, we demonstrated that Pb14-3-3 acts as an adhesin, even in a non-pathogenic model. This protein has adhesin function unlike Bmh1p, despite the high identity between these proteins.

The influence of Pb14-3-3 in ergosterol pathway was also demonstrated in this model and we believe that also happens in *Paracoccidioides* spp.. Further studies should be conducted in order to evaluate this new function of Pb14-3-3 and its implications in *Paracoccidioides* spp. pathogenesis and virulence. However, this highlights the importance of this protein and its potential as therapeutic target against *Paracoccidioides* spp.*.*

## Methods

### Microorganisms and growth conditions

In this study, *P. brasiliensis* Pb18 strain in the yeast phase was cultured in Fava Netto medium at 37 °C and was used to perform RNA extraction.

*S. cerevisiae wild type* (*wt*), *Δbmh1* (bmh1::KanMX4) and *Δbmh2* (bmh2::KanMX4) strains were generously provided by Dr. Cleslei Fernando Zanelli from the Molecular Biology Laboratory of Faculty of Pharmaceutical Sciences. They were maintained in YEPD (yeast extract peptone dextrose) medium at 25 °C. After transformation, the yeast cells were maintained in the selective medium SD-URA.

The *Escherichia coli* DH10B strain was employed for plasmid amplification and was maintained in Luria-Bertani (LB) medium. After transformation, the positive transformants were selected in LB supplemented with ampicillin (50 μg/mL).

### **Cloning of the***Paracoccidioides brasiliensis***14**-**3**-**3 gene into pYES expression vector**

Genomic DNA from *S. cerevisiae* strains were obtained through the phenol: chloroform: isoamilic alcohol method according to Hanna and Xiao [98], whereas the *P. brasiliensis* RNA extraction was performed by the TRIzol method (Invitrogen Life Technologies, Carlsbad, CA, USA) according to the manufacturer’s instructions followed by cDNA synthesis using reverse transcriptase (RevertAid™H Minus Reverse Transcriptase, Fermentas, Canada) and 1 μg of total RNA.

Amplification of Pb14-3-3 [GenBank: AY462124] and Bmh1p [GenBank: X66206.1] coding regions were carried out through PCR using the specific primers 14-3-3 F/14-3-3R and BMH1F/BMH2R (Table **1**). The PCR products were checked by agarose electrophoresis, purified using a QIAquick PCR Purification kit (Qiagen, Redwood City, CA, USA) and quantified using a NanoVue Plus (GE Healthcare Buckinghamshire, UK).

**Table 1:** Oligonucleotides used for plasmids construction and Real Time PCR study

Name	Description (5′–3′)
14-3-3F	CGGGATCCATGGGTTACGAAGATG
14-3-3R	CCGCTCGAGCTACTCAGCGGCCTTAGG
BMH1F	CG GGATCC ATGTCAACCAGTCGTGAAG
BMH1R	CG GAATTC TTACTTTGGTGCTTCACC
ERG1F	ATCCATTGACTGGTGGTGGT
ERG1R	CGGTCGCTGAAGTCTAGGTC
ERG11F	CCTCTTATTCCGTCGGTGAA
ERG11R	TGTGTCTACCACCACCGAAA
ERG28F	CAACCCATTTGAGTGCAAGA
ERG28R	GAAGTGGAATAGGGCAACCA
HES1F	TGTGGCAGAAGCAATCAGAC
HES1R	CTTTGCCATTCCACACCTTT
ACT1F	CGGTGATGGTGTTACTCACG
ACT1R	GGCCAAATCGATTCTCAAAA

The 14-3-3 amplicon was cloned into BamHI/XhoI sites of the pYES2 vector expression system (Invitrogen Life Technologies, Carlsbad, CA, USA), and the Bmh1 amplicon was cloned into BamHI/EcoRI sites of the pYES2 vector expression system (Invitrogen Life Technologies, Carlsbad, CA, USA). The obtained plasmids, pYES-14-3-3 and pYES-BMH1, were transformed into *E. coli* DH10B through the heat shock method, and then the bacteria was plated in LB medium supplemented with ampicillin 50 μg/mL and incubated overnight at 37 °C.

Positive colonies were grown, and plasmid extraction was performed using QIAprep Spin Miniprep (Qiagen, Redwood City, CA, USA). To ensure that the plasmid extracts were pYES-14-3-3 and pYES-BMH1, restriction diagnosis was performed using *HindIII* and *BamHI* enzymes (Promega, Madison, Wi, USA). After confirmation, transformation in yeast was initiated.

The *wt*, *Δbmh1* and *Δbmh2 S. cerevisiae* strains were submitted to yeast transformation with pYES-14-3-3 and pYES-BMH1 (used as a complementation control) through the lithium acetate method. After transformation, yeast cells were plated in SD-URA medium to select positive transformants and incubated at 30 °C until the appearance of growth.

### Spot test

The spot test was performed to evaluate the ability of Pb14-3-3 to complement the functions of Bmh1p or Bmh2p. Fluconazole was chosen after searching the *Saccharomyces* Genome Database, where a decrease in sensitivity to fluconazole was described for *S. cerevisiae Δbmh1* and *Δbmh2*.

Each transformant was grown in SD-URA medium until a concentration of 1×10^7^ cells/mL (A_600nm_ = 0.6–0.9), and suspensions of 2.5 × 10^8^ cells/mL were prepared in glycerol 50 %. Then, 100 μL of each sample was transferred to a 96-well plate, six serial dilutions were prepared and 2.5 μL of each suspension was spotted in SD-URA supplemented with 2 % galactose and 35 μM fluconazole (Sigma-Aldrich, St. Louis, MO, USA); control plates without fluconazole were also made. The plates were incubated at 30 °C until growth in all dilutions was observed in control plates. The experiment was conducted in triplicate with three independent experiments for each transformant.

### Expression analysis of ergosterol pathway genes *ERG1*, *ERG11*, *ERG28* and *HES1* by Real Time PCR

The evaluated genes, *ERG1*, *ERG11*, *ERG28* and *HES1*, were chosen according to Bruckmann et al. [26] and *ACT1* was used as housekeeping gene. Specific primers for each gene were synthetized (Table 1).

*Saccharomyces cerevisiae* transformants were grown in SD-URA medium overnight at 30 °C/150 rpm and then transferred to induction medium, which consisted of SD-URA with 2 % galactose. The cells were collected and RNA extraction was performed by the TRIzol method (Invitrogen Life Technologies, Carlsbad, CA, USA) according to the manufacturer’s instructions followed by first-strand cDNA synthesis using reverse transcriptase (RevertAid™H Minus Reverse Transcriptase, Fermentas, CA) and 1 μg of total RNA.

The reaction mixtures contained 1 μL of cDNA, 10 μL of Maxima ® SYBR Green/ROX qPCR Master Mix (2X) (Fermentas, Canada), 0.7 μM of each primer and nuclease-free water to the final volume of 20 μL The reactions were performed in Applied Biosystems 7500 Real Time PCR System (Applied Biosystems, Foster City, CA, USA) with the following program: 50 °C for 2 min, 95 °C for 10 min, and 40 cycles of 95 °C for 15 s followed by annealing and synthesis at 60 °C for 1 min. Following the PCR, a melting curve analysis was performed, which confirmed that the signal corresponded to a single PCR product. The data were analyzed using the 2^−ΔΔCT^ method. The cycle threshold values for the triplicate PCRs of each RNA sample were averaged, and then the values were calculated using the *ACT1* gene which was chosen as reference housekeeping gene [99].

Before the relative expression analyses, the efficiency of the amplifications were performed, then experiments were conducted in triplicate with three independent experiments for each primer and sample. Statistical analysis was performed using ANOVA test followed by Turkey’s post-test. The analyses and the graphs construction were conducted in GraphPad Prism5 software (GraphPad Software Inc., La Jolla, CA, USA).

### Adhesion assay

The adhesion assay was performed according to Younes et al. [100] with some modifications. *Saccharomyces cerevisiae* transformants were grown in SD-URA medium containing 2 % raffinose overnight at 30 °C/150 rpm and then transferred to induction medium (SD-URA with 2 % galactose) and incubated for 4 hours under the same conditions described above.

In 24-well plates, pneumocytes (ATCC A549) were plated with HAM-F12 medium supplemented with fetal bovine serum and incubated at 36.5 °C, 5 % CO_2_ until monolayer formation. Then, the cells were washed three times, and new medium was added. In each well, 300 μL of each inoculum was added to a final concentration of 1×10^4^ yeast cells/mL.

The plates were incubated for 30 min at 36.5 °C/5%CO_2_. After this period, they were washed to remove non adherent yeast cells. The epithelial cells were lysed with 100 μL of cold water and plated in YEPD medium incubated for 36 h/30 °C. The colony count was performed, and the adhesion rate was expressed as the percentage of adhered cells in relation to control plates.

The experiments were carried out in triplicate with three independent experiments and statistical analysis was performed using an ANOVA test with a Tukey’s post-test. The analyses and the graphs were conducted in GraphPad Prism5 software (GraphPad Software Inc., La Jolla, CA, USA).

## Abbreviations

ACO: aconitase
ATCC: American Type Culture Collection
ECM: extracellular matrix
ENO: enolase
FZ: fluconazole
GAPDH: glyceraldehyde-3-phosphate-dehydrogenase
Gp43: 43 kDa glycoprotein
ICL: isocitrate lyase
LB: Luria-Bertani
MLS: malate synthase
Pb14-3-3: 14-3-3 protein from *Paracoccidioides brasiliensis*
PCR: polymerase chain reaction
SD-URA: synthetic defined medium without uracil
TPI: triose phosphate isomerase
wt: wild type
YEPD: yeast extract peptone dextrose
